# Facilitators’ champion behaviors for delivering an evidence‐based parenting program

**DOI:** 10.1002/ajcp.70093

**Published:** 2026-08-12

**Authors:** Camila Regina Lotto, Elisa Rachel Pisani Altafim, Dana Charles McCoy, Maria Beatriz Martins Linhares

**Affiliations:** ^1^ Ribeirão Preto Medical School University of São Paulo São Paulo Brazil; ^2^ Harvard Graduate School of Education Cambridge Massachusetts USA

**Keywords:** ACT program, champion behaviors, facilitators, parenting program, resource availability, self‐regulation

## Abstract

Effective implementation of parenting programs relies on multiple factors, particularly the role of facilitators who actively deliver and support the intervention. Facilitators’ champion behaviors or the efforts they make to encourage implementation, can positively impact the outcomes of these programs. The present study examined the predictors of facilitators’ champion behaviors when delivering the ACT‐Raising Safe Kids Program in public services in an upper‐middle‐income country. The sample comprised 131 facilitators from 26 municipalities in Brazil. Participants completed self‐report questionnaires addressing sociodemographic and background characteristics, self‐regulation, resource availability, and champion behaviors. A partially latent structural equation model (SEM) was used to test direct and indirect relations between these variables. To support the SEM, we also conducted a confirmatory factor analysis (CFA) to assess the psychometric properties of the instruments. The results revealed that facilitators' self‐regulation and resource availability significantly and positively predicted facilitators' personal champion behaviors, but not their public behaviors. Facilitators’ self‐regulation served as a mechanism explaining the positive relation between resource availability and personal champion behaviors. The findings highlight the relevance of facilitators’ behaviors in delivering the program to families, as well as the importance of organizational resources for intervention implementation in public services.

## Highlights

- Organizational resources predicted self‐regulation and experience with program delivery.
- Organizational resources indirectly impacted personal champion behaviors.
- Self‐regulation mediated the relationship between resources and personal champion behaviors.

## INTRODUCTION

Parenting programs constitute an essential strategy for child development protection through strengthening parental knowledge, practices, and parent–child interactions and preventing violence against children (Branco et al., 2021; Jeong et al., 2021). However, the successful large‐scale implementation of parenting programs in real‐world settings faces several challenges (Pinto et al., 2024), including the need to support the individuals (Lansford et al., 2022) and the organizations involved with implementation (Murta et al., 2021).One effective strategy to support the impact of these programs that originate from implementation science is to ensure that facilitators—those responsible for program delivery—are carefully selected, thoroughly trained, provided with ongoing consultation throughout the implementation process, and regularly evaluated on their performance (Fixsen et al., 2009).

From a community psychology perspective, parenting interventions are embedded within broader cultural and ecological systems that influence families’ engagement and access to services (Trickett, 2009). Previous research has highlighted the importance of facilitators’ knowledge of local contexts, community resources, and available services, as well as organizational support and culturally representative staff, for successful implementation in community settings (Jürgensen et al., 2025). The selection of the core team of facilitators to deliver the program for families is crucial, as these individuals directly impact the viability and cost‐effectiveness of the program's implementation (Lansford et al., 2022). In this sense, “champion” facilitators are individuals who actively advocate for the intervention during its implementation (Damschroder et al., 2009). *Champions* act as active agents of change within organizations and have been identified as one of several key factors associated with successful program implementation (Damschroder et al., 2009; Miech et al., 2018). Therefore, facilitators’ champion behaviors may contribute to strengthening community engagement and increasing access to evidence‐based parenting interventions.

The Consolidated Framework for Implementation Research (CFIR), proposed by Damschroder et al. (2009), highlights the critical role played by champions in supporting program implementation. According to CFIR, leaders, who may occupy formal or informal positions, are responsible for mobilizing support, engaging colleagues, overcoming resistance, and maintaining focus on the intervention's goals. CFIR positions them in the “process” domain, highlighting their strategic role in complex and dynamic organizational contexts, especially when the adoption of innovation requires behavioral and cultural changes (Damschroder et al., 2009, 2022). Therefore, a champion can take on multiple roles within an organization, actively leading the implementation process, fostering its uptake, and serving as a connector between various stakeholder groups, whether formally appointed by the institution or not (Shea, 2021). Specifically, champion facilitators promote the program in their personal and professional relationships with program users, supporting the initiative (Ma, Tellegen, & Sanders, 2023). Thus, having at least one champion is a valuable resource for the implementation and sustainability of the program (Durlak & DuPre, 2008). Moreover, champions can contribute during the sustainment phase by monitoring the use of innovation, addressing participants' needs, and mitigating potential barriers to ongoing implementation (Shea, 2021).

According to Ma, Tellegen, and Sanders (2023), *champion behaviors* can be understood as the specific set of proactive and advocacy‐oriented actions undertaken by individuals who actively support and promote evidence‐based initiatives (e.g., program facilitators). These champion behaviors can be categorized into two types: public and personal. *Public champion behaviors* involve actions such as speaking publicly to encourage participation and sharing information about the program through social media, television, or radio. *Personal champion behaviors*, on the other hand, occur at the individual level and include recommending the program to colleagues, families, and service users, actively advocating for the program, and defending it against criticism (Ma, Tellegen, & Sanders, 2023).

Previous studies have examined champion behaviors within the Triple P parenting program in the United States, Canada, Australia, and the United Kingdom, using validated instruments (Ma, Tellegen, & Sanders, 2023; Turner & Sanders, 2006). Other research has investigated how community champions and key stakeholders mobilize knowledge and support parenting programs across diverse contexts, but without employing a specific instrument to assess champion behaviors (Coore‐Hall et al., 2023; Wilkinson et al., 2024). Therefore, to our knowledge, no research has examined facilitators’ champion behaviors using a validated measure in parenting programs implemented in low‐ and middle‐income countries (LMICs), where resources and cultural norms may differ.

### Predictors of facilitators’ champion behaviors

Given the potential benefits of champion behaviors for improving program implementation outcomes, it is important to understand precisely what factors encourage facilitators to behave as champions. Previous research about the Triple P program has identified relevant predictors of facilitators’ champion behaviors (Ma, Tellegen, & Sanders, 2023). In the present study, we build on this work to consider three possible factors associated with champion behaviors: self‐regulation, facilitators’ experience with program delivery, and organizational resources.

First, *self‐regulation* could be an important predictor of facilitators’ champion behaviors. Self‐regulation can be understood as the capacity to monitor and regulate emotional, cognitive, and behavioral processes to achieve a goal or adapt to specific situational demands (Berger et al., 2007). Self‐regulation plays a central role in parenting interventions and is a core competency for parents and facilitators, enabling them to adapt their behavior in the face of difficulties and to promote adaptive self‐management strategies among parents (Sanders & Mazzucchelli, 2013; Tellegen et al., 2022). In this context, self‐regulation refers to facilitators’ ability in the implementation of a program, to plan, monitor, solve problems, and maintain motivation to deliver the program (Tellegen et al., 2022). Parenting programs can enhance self‐regulation skills for both parents and facilitators (Sanders & Mazzucchelli, 2013). Furthermore, the facilitators play a crucial role in helping parents develop self‐regulation skills by fostering supportive relationships and serving as role models (Sanders & Mazzucchelli, 2013; Sanders et al., 2019). Therefore, assessing facilitators’ professional self‐regulation should be an important component of implementation studies. When facilitators have more self‐regulation skills in their parenting program delivery, it can influence their beliefs and attitudes, increasing their motivation to advocate for the program (Ma, Tellegen, & Sanders, 2023). Indeed, in a previous study involving another parenting program, practitioners’ self‐regulation skills were found to mediate the relationship between organizational support and their champion behaviors (Ma, Tellegen, & Sanders, 2023).

Second, the *facilitators’ experience with program delivery* may also influence the likelihood of champion behaviors. In a previous study on the champion behaviors of Triple P practitioners, there was a positive correlation between the experience with program delivery and champion behaviors (Ma, Tellegen, & Sanders, 2023). Accordingly, there is still a need to examine facilitators’ experience with program delivery in different implementation contexts, as its influence could vary depending on organizational structure and resources, facilitator roles, and program characteristics.

Third, broader organizational factors, including the resources provided to facilitators to complete their work, are also likely to enable champion behaviors and, in turn, implementation (Shapiro et al., 2012). *Organizational resources* are crucial for community‐based programs (Berry et al., 2019) and include both tangible supports, such as space for program implementation and preparation, and financial investment for participant incentives (Asgary‐Eden & Lee, 2012; Lansford et al., 2022; Menezes et al., 2020; Turner & Sanders, 2006), as well as less tangible supports such as time and encouragement to integrate program activities into facilitators’ routines and responsibilities (Durlak & DuPre, 2008; Shapiro et al., 2012). Conversely, a lack of organizational resources, such as inadequate infrastructure, competing priorities, and limited finances, creates barriers to facilitators' champion behaviors (Murta et al., 2021; Osman et al., 2022).

Importantly, organizational resources may support facilitators’ champion behaviors not only directly but also through indirect pathways, such as the professionals’ own self‐regulatory capacity in implementation, satisfaction with program resources, and perceived usefulness (Ma, Tellegen, & Sanders, 2023). These resources can shape beliefs and attitudes, sustaining motivation over time. Thus, organizations must provide the necessary support for facilitators to develop as champions (Ma, Tellegen, & Sanders, 2023). Nevertheless, the precise ways in which organizational resources may support champion behaviors remain understudied, particularly within LMICs (Shea, 2021).

### Parenting program implemented in Brazil: The ACT—Raising Safe Kids Program

Although a limited body of research—primarily conducted in high‐income contexts using the Triple P program—has explored how to measure and predict facilitators’ champion behaviors, these processes have received little attention in the context of LMICs. Moreover, no studies have analyzed the relationship between champion behaviors, self‐regulation, experience, and organizational resource availability—key variables for facilitating program implementation in LMICs.

Recent studies have shown lifelong detrimental outcomes for children who suffer physical punishment in LMICs, underscoring the need for additional research into parenting interventions in these contexts (Cuartas et al., 2025). Violent forms of discipline, including physical and psychological aggression, remain prevalent in many LMICs (Cuartas et al., 2019), and their effects are often compounded by broader social vulnerabilities that increase the risk of maltreatment against children (Cerna‐Turoff et al., 2021). In Brazil, for example, there is legal protection of children, such as the Statute of Child and Adolescent (Brasil, 1990) and Law No. 13,010 (Brasil, 2014), which prohibit physical punishment and cruel or degrading treatment for children. However, despite this legal framework, a high proportion of caregivers in Brazil still believe that physical punishment is necessary (Venancio et al., 2022). These beliefs are embedded in broader parenting values and practices shaped by the country's cultural, regional, and socioeconomic diversity (Tudge et al., 2018). Such values often emphasize autonomy, obedience, proper presentation, and relatedness, although the importance attributed to these aspects varies across different regions of Brazil (Tudge et al., 2018). For example, within this broader context, some Brazilian religious groups interpret the use of physical punishment as a legitimate method of child‐rearing rather than as a form of violence against children (Deslandes et al., 2023).

At the same time, recent global analyses highlight that LMICs are home to the majority of the world's children and that early childhood development research and parenting interventions remain concentrated in high‐income contexts (Draper et al., 2023, 2024). There is a need for evidence to inform practice and strengthen the effectiveness of violence prevention interventions in these contexts (Shenderovich et al., 2021). Moreover, the successful scale‐up of such interventions in LMICs faces substantial challenges, including the need for multi‐sectoral cooperation, resource constraints, and the complexities of adapting programs to local systems and contexts (Shenderovich et al., 2021). Therefore, there is an urgent need to develop structured parenting programs in this context, as well as to study the factors that encourage their implementation and the effectiveness of parenting programs on a large scale. The present study addresses these gaps by focusing on the parenting program named ACT‐Raising Safe Kids Program in Brazil (ACT derives from the English word act (to take action) and is the official name of the program). The ACT Raising Safe Kids Program is a preventive, universal, evidence‐based parenting psychosocial intervention designed to strengthen positive parenting, including emotional and behavioral self‐regulation practices, and to prevent violence against children (Silva, 2011; American Psychological Association [APA]; Altafim et al., 2024). The program is implemented in several countries, including Brazil, and is grounded in social learning theory, cognitive‐behavioral principles, and developmental theory (Silva, 2011). In Brazil, the ACT program was effective in improving mothers’ parenting practices (e.g., positive discipline, communication, and emotional regulation) and reducing children's behavior problems with different samples (Altafim & Linhares, 2019; Altafim et al., 2024). Also, the program was implemented effectively on a large scale in the public services of Brazil, across 17 municipalities in the state of Ceará (Altafim & Linhares, 2025). This initiative prioritized socially vulnerable families, particularly those receiving income transfer benefits. Implementation was conducted by trained and certified facilitators—psychologists, social workers, and educators—from public social services, ensuring fidelity and quality in delivering the program (Altafim & Linhares, 2025). Importantly, ACT Program training guidelines recommend that facilitators have prior experience with structured programs or with families, as such experience can enhance training participation and implementation quality (American Psychological Association, 2026). However, there remains a gap in research concerning specific facilitator behaviors that can support large‐scale program implementation (Altafim et al., 2024).

Furthermore, community psychology in Latin America emphasizes participation, prevention, and social transformation (Montero, 2008). Recent analyses of the field have also highlighted the importance of expanding knowledge production from underrepresented regions, including Latin America (Perkins et al., 2023). In Brazil, this field was linked to social movements and reforms in the mental health area (Gonçalves & Portugal, 2016). These principles are highly relevant to parenting interventions, which aim to prevent violence against children, strengthen family resources, and promote positive developmental environments (Altafim et al., 2024). From a community psychology perspective, facilitators may contribute to strengthening connections between families, communities, and public service systems (Jürgensen et al., 2025). Furthermore, they can support community engagement and facilitate the alignment of parenting programs with local needs, resources, and existing infrastructures (Lansford et al., 2022). Therefore, understanding the factors that support facilitators’ champion behaviors may contribute to both implementation science and community psychology.

### The present study

The main objective of the present study was to examine the predictors of the facilitators’ champion behaviors within the ACT‐Raising Safe Kids program implemented in public services in Brazil. In particular, we examined facilitators' self‐regulation skills, experience with program delivery, and the availability of organizational resources during the program implementation as potential predictors of facilitators’ public and personal champion behaviors. In addition to exploring the direct associations of these variables with champion behavior, we also investigated the indirect ways that organizational resources may shape facilitators’ behavior outcomes. In particular, building on previous research, we hypothesized that organizational resource availability enhances facilitators’ self‐regulation behaviors, leading to a greater likelihood of facilitators exhibiting champion behaviors. Furthermore, we hypothesized that the facilitators’ experience with program delivery with families and self‐regulation skills would positively predict their champion behaviors. To our knowledge, this combination of predictors has not been examined in previous champion behavior studies in the context of the ACT Program. Furthermore, as far as we know, this is the first study to consider predictors of champion behaviors within an LMIC context. Accordingly, the goal of this study is to inform the development of approaches that may enhance champion behaviors and, in turn, intervention effectiveness in underrepresented LMICs.

## METHOD

### Sample study

The sample comprised 131 participants, who were professionals (e.g., social workers, psychologists, and teachers) working with children and families in public services (e.g., social protection and educational services). The inclusion criteria required participants to have completed the ACT Program training and received certification as ACT facilitators. The professionals were from 26 municipalities in Brazil's Northeast (Ceará State) and Southeast (São Paulo and Minas Gerais States) regions. Of the 168 certified ACT facilitators, we could not reach four because their phone numbers were no longer in service. Of the 164 facilitators who were contacted, 33 did not respond to the survey. Specifically, 2 declined to participate, and 31 did not respond to our attempts to contact them. Consequently, the final sample included 131 facilitators who completed the questionnaires, yielding a response rate of 78%.

### Ethical aspects

The Research Ethics Committee of the Clinical Hospital of the Ribeirao Preto Medical School of the University of São Paulo approved the study. All participants signed an Informed and Free Consent Form before the data collection.

### The ACT program training

The ACT—Raising Safe Kids Program (Silva, 2011) (Brazilian‐Portuguese version), developed by the APA, is an evidence‐based program delivered in groups for caregivers of children up to 8 years old, aiming to develop positive parenting practices and prevent violence. The program includes structured materials for conducting nine highly interactive sessions delivered weekly: one pre‐meeting and eight intervention sessions, each lasting approximately 120 min. The sessions include several activities, such as small group discussions, role‐plays, practical exercises, homework assignments, facilitators’ explanations, and video presentations.

According to the American Psychological Association (2026), to participate in the training and receive certification as an ACT Program facilitator, professional candidates must meet the following requirements: hold a minimum of an associate's degree, but a bachelor's degree is preferred; demonstrate organizational support to implement the program; have previous experience conducting groups; and have general knowledge of child development. The ACT facilitator certification process consisted of two phases: participating in a 16‐h theoretical training (online or in‐person) and performing practical training by conducting a group session with families. The theoretical training addressed facilitators’ behavior, motivational strategies to promote parenting changes, program curriculum and activities (e.g., role‐playing and dynamics of groups), and implementation procedures. Importantly, many of the activities that parents later experience in their groups are first practiced during this theoretical training, allowing facilitators to engage with the material from the participants’ perspective. These training components emphasize emotional and behavioral regulation, particularly the management of anger and other feelings that may arise during program delivery. Following the theoretical phase, facilitators underwent practical training, which involved leading a group with parents and recording one session for evaluation. These videos were evaluated, and once approved by master trainers, the facilitators received their certification as ACT facilitators (American Psychological Association, 2026; for more information, see the ACT website) https://www.apa.org/act).

### The ACT program implementation

Initially, each municipality nominated professionals to participate in the training and certification process for the ACT program. Once certified, the facilitators implemented the program within social protection services or schools in their respective municipalities. This implementation was conducted in collaboration with municipal governments and was incorporated into the facilitators’ regular working hours. Program participants were beneficiary families of public social protection services or parents of school‐attending children. In addition, the organizational support was the responsibility of the municipalities and the respective services implementing the program.

### Procedures

#### Data collection

Data collection occurred from July to September 2024. The facilitators were invited to participate in the study via phone calls or messages. We scheduled a time for those who responded to complete the questionnaires by phone. At the beginning of data collection, we explained all procedures to the facilitators and obtained free consent to participate in the study. Of the facilitators who participated, 93 completed the questionnaires by phone with the first author, while 38 preferred to receive a link to complete the questionnaires online at their convenience.

#### Instruments and measures

##### Questionnaire about facilitators' sociodemographic and background characteristics

The questionnaire included information on the following variables: facilitator age, sex (male *vs*. female), type of bachelor's degree (e.g., Social Assistance, Psychology, Education Teacher, or others), time in public service (up to 1 year, from 2 to 5 years, from 6 to 10 years, and 11 years or upper), conducting groups with families (up to 1 year, from 2 to 5 years, from 6 to 10 years, and 11 years or upper), and previous experience in implementing structured programs with families before the ACT Program training (yes *vs*. no).

##### Question item on ACT Program delivery experience

The item assessed the facilitators' experience with program delivery. Facilitators were asked to indicate the number of groups they had facilitated within the ACT Program, choosing from three response options: only the certification group, two to five groups, or more than six groups.

##### Parenting Self‐Regulation Scales—Practitioner version

Facilitators' professional self‐regulation skills were measured using the Parenting Self‐Regulation Scales—Practitioner version (PSRS—Practitioner version, Sanders et al., 2017; Tellegen et al., 2022). The scale was translated and back‐translated into the Brazilian‐Portuguese language, authorized by the authors (Linhares & Altafim, 2022). This scale is an adaptation of the Parenting Self‐Regulation Scales—Parent Version (PSRS‐Parent, Sanders et al., 2017), adapted for practitioners rather than parents (Sanders et al., 2017). It is designed for use with professionals trained in any parenting program. It consists of 12 items rated on a 7‐point Likert‐type scale, ranging from 1 (“Strongly Disagree”) to 7 (“Strongly Agree”), with all items worded positively. Some examples of items are the following: “I know what behaviors and skills I want to encourage in parents”; “I feel confident that I can take actions to help parents improve their parenting behavior”; “I have the skills to manage problems that parents have with their parenting behavior or emotions”. Scores were calculated by summing the items, with higher total scores indicating greater levels of self‐regulation among the professionals. The current study inserted the name of the ACT program in items where indicated.

##### The Resource Availability Scale

The Resource Availability Scale was adapted from the Questionnaire on Professional Knowledge for Guiding Parents and Caregivers, developed by Linhares and Altafim (2017) and previously used in the study by Linhares et al. (2017). This scale assesses facilitators’ perceptions of the organizational availability of resources within the public services, including time, staff, a place for conducting groups, training materials, technological equipment, and organizational support. Responses were rated on a Likert scale from 1 to 4 (“Very Few” = 1;” Few” = 2; “Some” = 3; “Very” = 4).” Higher scores mean greater availability of resources in the public services.

##### Champions Behavior Scale

Facilitators’ champion behaviors were measured by the Champions Behavior Scale (Ma, Tellegen, & Sanders, 2023), translated and back‐translated into the Brazilian‐Portuguese language authorized by the authors (Linhares & Altafim, 2022). This scale identifies 13 potential exemplary behaviors (champion behaviors) that a facilitator may have exhibited in the past 12 months. It comprises 13 items, and factor analysis by Ma, Tellegen, and Sanders (2023) revealed two subscales: Public Champions’ Behavior (seven items; e.g., “I speak in public (e.g., schools) to encourage parents to participate in the ACT Program”; “I personally appear on TV or radio to discuss the program”) and Personal Champions’ Behavior (six items; e.g., “I defend the ACT Program against criticism”, “I recommend the ACT Program to friends, family members or acquaintances). Responses were rated on a 4‐point Likert scale, ranging from 0 (“Disagree”) to 3 (“Strongly Agree”). A total score was calculated by summing the items, with higher scores indicating higher levels of champion behavior among the facilitators. In the current study, the name of the ACT Program was incorporated into questionnaire items where the program's name was required.

## ANALYTIC APPROACH

### Data analysis

Regarding missing values, only one participant did not respond to one item (technology equipment) in the Resource Availability Scale. We substituted the missing value with the mean of that item for others in the sample.

To address the research questions of the current study, we conducted a structural equation model (SEM), including measurement and structural components. Before fitting the SEM, we first tested the measurement model of each latent variable—facilitators’ self‐regulation (using the PSRS‐Practitioner Version), organizational resource availability (using the Resource Availability Scale), and facilitators’ champion behavior (using the Champion Behavior Scale)–separately, using confirmatory factor analysis (CFA) to verify whether the factor loadings and model fit were acceptable (Kline, 2023). Based on the previous research study (Tellegen et al., 2022), a one‐factor model was tested for facilitators’ self‐regulation. A one‐factor model was also tested for resource availability, including the available time, staff, a place to conduct groups, training materials, technology equipment, and organizational support. Finally, for champion behavior, we tested a two‐factor structure proposed by the scales’ developer (Ma, Tellegen, & Sanders, 2023). A one‐factor model for Champion Behavior was also tested; however, model comparison using a chi‐square difference test indicated that the two‐factor model provided a better fit and aligned with findings from previous research (Ma, Tellegen, & Sanders, 2023). Accordingly, the two‐factor model was retained, consistent with theoretical, conceptual, and empirical rationales supporting this structure. These CFAs were conducted with the full sample (*n* = 131).

In this study, items were removed during the CFA based on low factor loadings (<0.4), as recommended by Hair et al. (2014), and guided by theoretical and contextual relevance to preserve the integrity of the construct. Additionally, we compared the model fit with and without the deleted items to assess whether their exclusion improved overall fit. After achieving a well‐fitting model for each instrument, a partially latent SEM was conducted to test direct and indirect relations between available organizational resources, facilitators’ self‐regulation, ACT Program delivery experience (number of ACT groups facilitated), with their champion behavior. We included resource availability as a predictor variable, the facilitators’ self‐regulation and ACT Program delivery experience as mediator variables, and the facilitators’ champion behavior as the outcome. The facilitators’ prior experience with structured programs with families was the only covariate included, based on recommendations from the ACT Program training. These guidelines suggest that selecting facilitators who already have experience with structured programs or working with families may be beneficial for training participation (American Psychological Association, 2026). Accordingly, this covariate was included in the SEM model with direct paths to both the outcome and the mediator variables.

Model fit was examined for both the CFAs and full structural model, using four indices: (i) a relative *χ* ^2^ value (the ratio of *χ* ^2^ to degrees of freedom) of 3 or less, (ii) a root‐mean‐square error of approximation (RMSEA) of less than 0.08, (iii) a standardized root mean square residual (SRMR) of less than 0.08, and (iv) a comparative fit index (CFI) of 0.90 or above (with > 0.95 considered ideal; Hatcher, 1994; Hu & Bentler, 1999; Kline, 2023). The model was fit using the maximum likelihood (ML) estimator. Additionally, Cronbach's *α* were calculated to evaluate the internal consistency of each latent variable, with an *α* above 0.60 considered acceptable (Loewenthal, 2001).

Descriptive analysis and database construction were performed using the Statistical Package for Social Sciences (SPSS, version 23.0; IBM, IL, Chicago, USA). CFA and SEM analysis were conducted using Stata Statistical Software (STATA version 13.0). Results were considered statistically significant at *p* ≤ .05.

## RESULTS

### Sample characteristics

According to Table 1, the facilitators were predominantly women, with most having worked in public service for over 2 years under temporary contracts. Additionally, nearly half reported having experience in implementing structured programs with families.

Additionally, regarding facilitators’ experience in conducting groups, most facilitators conducted two to five groups (*n* = 82, 63%), while the remaining facilitators either conducted only the certification group (*n* = 38, 29%) or more than six groups (*n* = 11, 8%).

**Table 1:** Sociodemographic and background characteristics of the sample (*n* = 131).

Sociodemographic and background characteristics of the sample	Values
Sociodemographic characteristics	
Age (years)—mean (SD; range)	39 (± 10; 23 – 67)
Sex ‐ *n* (%)	
Female	117 (89)
Male	14 (11)
Bachelor's degree—*n* (%)	
Social Service	52 (40)
Psychology	39 (30)
Teacher Education	24 (19)
Others	16 (11)
Background characteristics	
Type of contract—*n* (%)	
Temporary	78 (60)
Government employees	42 (32)
Others	11 (8)
Time in service—*n* (%)	
Up to 1 year	43 (33)
From 2 to 5 years	55 (42)
From 6 to 10 years	10 (8)
11 years or upper	23 (17)
Time of experience in conducting groups with families—*n* (%)	
No previous experience	32 (24)
Up to 1 year	25 (19)
From 2 to 5 years	33 (26)
From 6 to 10 years	21 (16)
11 years or upper	20 (15)
Previous experience in implementing structured programs with families—*n* (%)	
Yes/No	62 (47)/69 (53)

Abbreviations: *n*, number of participants; %, percentage; SD, standard deviation.

## MEASUREMENT MODELS

### Parenting self‐regulation scales—practitioner version

**Figure 1:**
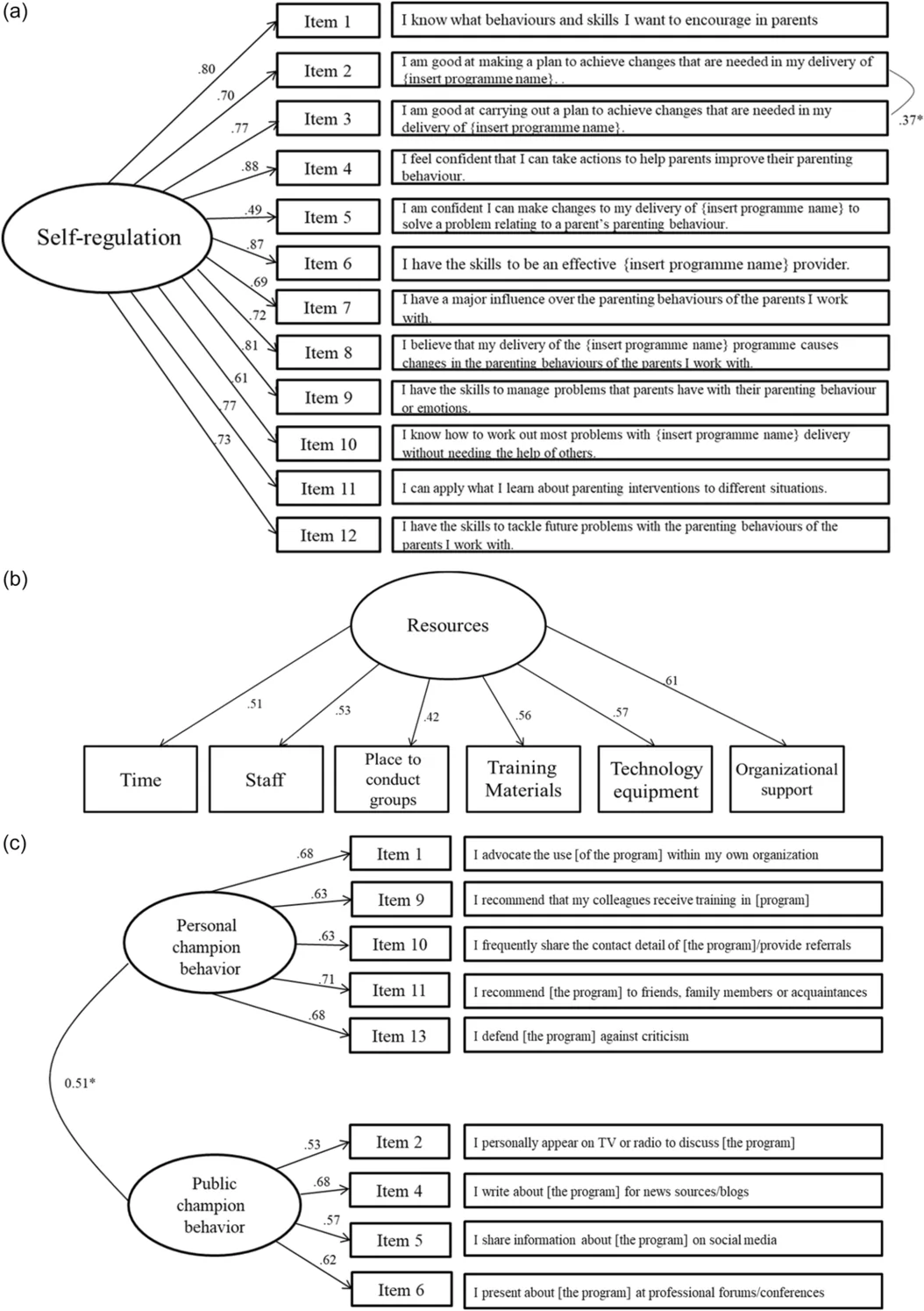
Measurement models. Full sample (*n* = 131). Factor loadings are standardized. All factor loadings are significant at *p* < .001. (a) Parenting Self‐Regulation Scales—Practitioner version. (b) Resource Availability Scale. (c) Champion Behavior Scale.

A one‐factor model of facilitator self‐regulation showed mostly adequate fit (*χ* ^2^/*df* = 2.22; CFI = 0.93; SRMR = 0.04), with RMSEA showing an elevated value of 0.097. Based on findings from a previous psychometric study of the Parenting Self‐Regulation Scale (Tellegen et al., 2022), we added a residual covariance between two items (items 2 and 3, “I am good at making a plan to achieve changes that are needed in my delivery of the ACT Program” and “I am good at carrying out a plan to achieve changes that are needed in my delivery of the ACT Program”). After this modification, the overall model was considered adequate (*χ* ^2^/*df* = 1.94; RMSEA = 0.086; CFI = 0.95; SRMR = 0.04), comprising 12 items, and all factors loading at least 0.4, and an internal consistency of 0.93 (see Figure 1a).

### The resource availability scale

The one‐factor model of organizational resources showed an adequate overall model fit (*χ* ^2^/*df* = 1.65; RMSEA = 0.07; CFI = 0.94; SRMR = 0.05). All six items had loadings of at least 0.4, and the internal consistency was 0.70 (see Figure 1b).

### Champion Behavior Scale

We tested the two‐factor model for facilitator champion behavior based on the previous study by Ma et al. (2022). The initial model did not fit adequately (*χ* ^2^/*df* = 1.89; RMSEA = 0.08; CFI = 0.86; SRMR = 0.09). Consequently, items with loadings below 0.40 were removed: one item from the personal champion behavior factor (12 “I direct parents or peers to the ACT Program website”) and two items from the public champion behavior factor (7 “I speak in public (e.g., schools) to encourage parents to participate in the ACT Program”, and 8 “I apply for funding or grants relating to the ACT Program”). Item 3 from the public champion behavior factor was removed based on content considerations, as it is not a behavior related to implementation, especially in the public context (“I write journal articles or research papers/reports about the ACT Program”). The final two‐factor model demonstrated an overall adequate fit (*χ* ^2^/*df* = 1.76; RMSEA = 0.07; CFI = 0.93; SRMR = 0.07), comprising five items for the personal factor and four items for the public factor. All factor loadings were at least 0.4. The internal consistency was 0.78 for personal champion behavior and 0.67 for public champion behavior (see Figure 1c).

### Structural equation model for explaining the facilitators’ champion behaviors

**Figure 2:**
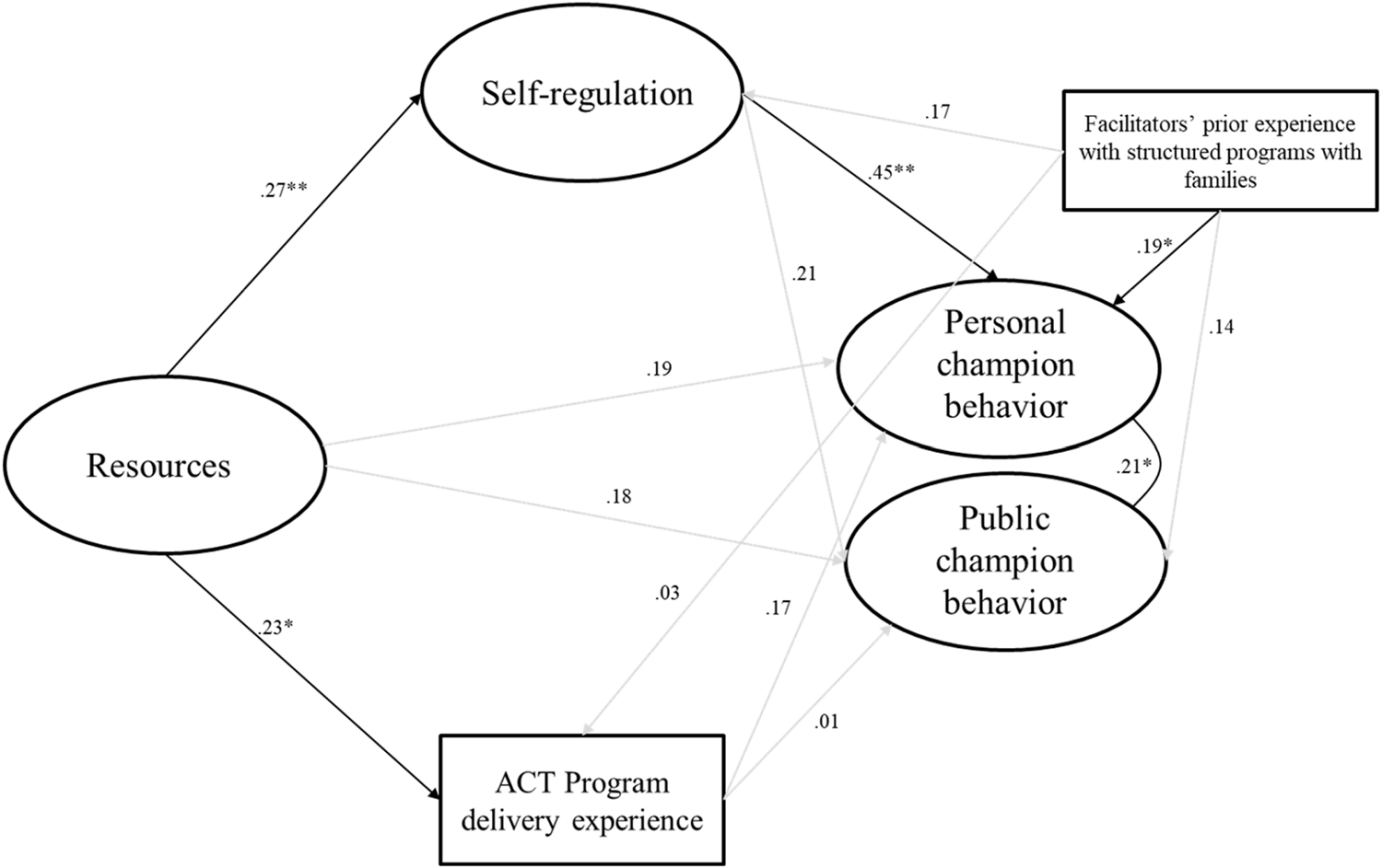
Structural Equational Model of the facilitators’ personal and public champion behaviors with covariate. Full sample (*n* = 131). *p < .05. **p < .01. The black lines represent significant pathways, and the gray lines represent nonsignificant pathways. The indirect effect of resource availability on personal champion behaviors through self‐regulation was significant. Rectangles represent the observed variables, and a circle represents the latent variables. All latent variables are embedded measurement models (for resources, self‐regulation, and champion behavior) included in the model but not shown (see Figure 1 to view the measurement models). Standardized coefficients are described.

The full structural model examining predictors of facilitators’ champion behaviors revealed adequate overall model fit (*χ* ^2^/*df* = 1.41; RMSEA = 0.06; CFI = 0.90; SRMR = 0.07). It also revealed several significant direct pathways (see Figure 2; Table 2). Regarding the direct effects, first, organizational resource availability was a significant positive predictor of the ACT Program delivery experience and facilitators’ self‐regulation. However, organizational resources did not directly predict either type of facilitator champion behaviors. Second, facilitators’ self‐regulation was statistically significant and positively predictive of personal champion behaviors, but not their public champion behaviors. The ACT Program delivery experience was not significantly associated with either personal or public champion behaviors. Regarding the indirect pathways, we identified a significant indirect path between resource availability and personal champion behaviors through self‐regulation (*b* = 0.09 [SE = 0.04]; *p* = .03). No other indirect paths were statistically significant.

**Table 2:** Results of partially latent structural equation model for explaining facilitators’ champion behaviors.

Variables	*b*	SE	*beta*
Resources → ACT delivery experience	0.26*	0.12	0.23
Resources → self‐regulation	0.51**	0.22	0.27
Resources → personal champion behaviors	0.14	0.08	0.19
Resources → public champion behaviors	0.10	0.08	0.18
Self‐regulation → personal champion behaviors	0.18**	0.04	0.45
Self‐regulation → public champion behaviors	0.06	0.03	0.21
ACT delivery experience → personal champion behaviors	0.11	0.05	0.17
ACT delivery experience → public champion behaviors	0.007	0.05	0.01
Previous experience → personal champion behaviors	0.14*	0.06	0.19
Previous experience → public champion behaviors	0.08	0.06	0.14
Previous experience → self‐regulation	0.33	0.17	0.17
Previous experience → ACT delivery experience	0.03	0.10	0.03
Resources → self‐regulation → personal champion behaviors	0.09*	0.04	0.12

Abbreviations: *b*, unstandardized coefficient; SE, standard error; *beta*, standardized coefficient.

* *p* ≤ .05

** *p* ≤ .01.

## DISCUSSION

To the best of our knowledge, this is the first study to investigate the personal and public champion behaviors of parenting program facilitators, who were professionals at the frontline of public services in a middle‐income country. The facilitators were serving as ACT‐Raising Safe Kids Program implementers and working with families in social protection services in 26 Northeast and Southeast Brazil municipalities. Using structural equation modeling, we found that facilitators’ self‐regulation skills and resource availability significantly and positively predicted their personal champion behaviors, but not their public behaviors. Importantly, resource availability predicted facilitators’ personal champion behavior indirectly through improving their self‐regulation.

The current study adds to the findings of the literature on facilitators’ champion behaviors and self‐regulation skills in the context of the ACT Program, which focuses on supporting parents’ self‐regulation and use of positive disciplinary approaches. As an initial step, we examined the psychometric properties of measures of champion behaviors and their possible predictors. These measurement results suggest that it is possible to capture facilitators’ self‐regulation, the availability of organizational resources, and facilitators’ champion behaviors using self‐reported measures implemented with public service professionals in Brazil. These findings are aligned with previous studies with Triple P implemented in several contexts (Ma, Tellegen, & Sanders, 2023; Tellegen et al., 2022).

Our results also suggest that organizational resource availability is a key factor that may support the well‐being and behavior of parenting program facilitators. Specifically, facilitators’ self‐regulation was positively predicted by organizational resource availability. This finding is aligned with previous studies of another parenting program that identified organizational support as a predictor of facilitator self‐regulation in delivering the Triple P program (Ma, Tellegen, & Sanders, 2022, 2023). Facilitators who demonstrate high self‐regulation are better equipped to model effective practices, fostering a supportive environment that encourages parental engagement and skill development (Sanders & Mazzucchelli, 2013). Furthermore, organizational resource availability was also found to directly shape facilitators’ experience with program delivery. These results underscore the importance of organizations providing adequate resources for the effective implementation of parenting programs, as this variable directly predicts both the facilitators’ characteristics and their experiences with group facilitation (Berry et al., 2019; Durlak & DuPre, 2008; Gwebu et al., 2024; Jürgensen et al., 2025; Turner & Sanders, 2006).

Our study also demonstrates that organizational resource availability can indirectly predict facilitators’ personal champion behaviors through supporting facilitators’ self‐regulation. This is similar to the findings from the Triple P program in high‐income countries found in Ma, Tellegen, and Sanders (2023). Unlike previous studies, our research highlights that resource availability extends beyond organizational support, highlighting other resources’ importance in personal champion behaviors, such as time allocated to deliver the program, appropriate spaces for group sessions, training materials, and technological equipment. In turn, self‐regulation skills, as highlighted in the literature, may foster facilitators’ positive attitudes and motivation to advocate for the program (Ma, Tellegen, & Sanders, 2023). Furthermore, it is worth noting that the self‐regulation skills examined in this study refer to the ability to plan, monitor progress, solve problems, and sustain participant motivation throughout program delivery (Tellegen et al., 2022). Our findings suggest that these skills are associated with personal champion behaviors, which are more closely linked to individual‐level actions such as recommending the program and advocating for its use (Ma, Tellegen, & Sanders, 2023). Organizations must support facilitators by providing the necessary conditions for them to become champions (Ma, Tellegen, & Sanders, 2023), as facilitators’ personal champion behaviors—such as advocating for the program and recommending it to friends, colleagues, and family members—can enhance implementation outcomes by fostering a supportive environment that promotes adoption and user engagement (Shea, 2021). Also, in the Brazilian context, facilitators may contribute to strengthening connections between families, public services, and evidence‐based parenting practices. This interpretation is consistent with community psychology perspectives emphasizing social change, public policies, and collective practices (Gonçalves & Portugal, 2016; Montero, 2008; Perkins et al., 2023).

Consistent with previous findings (Ma, Tellegen, & Sanders, 2023), our study also found no direct association between facilitators’ experience with program delivery and either type of champion behavior. This supports the idea that advocacy for a program often reflects a deeper level of commitment than is required by one's formal responsibilities, as proposed for Ma, Tellegen, and Sanders (2023). As shown in the previous study, facilitators are expected to deliver the program, not necessarily to promote or advocate for it (Ma, Tellegen, & Sanders, 2023). As such, simply improving facilitators’ experience with the program is unlikely to support their champion behaviors.

The present study found no significant association between public champion behavior and facilitators’ experience with program delivery, self‐regulation skills, or organizational resources. This is a relevant finding, as it suggests that other factors beyond individual competencies, actions, or contextual support may influence public champion behaviors. As highlighted in a systematic review study, facilitators can have different roles—for instance, some may act as coordinators responsible for logistical tasks, while others serve as implementation facilitators (Jürgensen et al., 2025). In this sense, public champion behaviors appear to align more closely with coordination roles rather than with implementation roles, which were the focus of the present study's sample. Further research is needed to better understand the mechanisms associated with public champion behavior within the Brazilian context.

The current study has several practical implications for implementing parenting programs in public services. First, we tested three fast and easy‐to‐use instruments to assess resource availability and two types of facilitators' behaviors that support the implementation of parenting programs, providing valuable instruments for future research and practice. Second, our findings highlight the importance of organizational resource investment to facilitate the effective implementation of parenting programs. By recognizing the significant role of resource availability for predicting facilitators’ behaviors, such as self‐regulation, organizations can better support their staff and enable facilitators to perform their roles effectively. Finally, identifying these behaviors is crucial for providing insights to organizations involved in parenting programs, enabling them to offer essential support in training and supervising facilitators and implementing the program within the public policy framework of an upper‐middle‐income country like Brazil.

The association between self‐regulation, organizational resources, and personal champion behaviors may be particularly relevant in the Brazilian context, where parenting programs are commonly delivered by professionals working within public service systems and local communities. A previous implementation study of the ACT Program in Brazil demonstrated that professionals from local services successfully implemented the program with high fidelity across multiple municipalities (Altafim et al., 2026). In this context, personal champion behaviors may represent an important mechanism through which facilitators engage families, promote access to evidence‐based parenting resources, and support the dissemination and sustainability of interventions within community settings.

The present study has some limitations. First, it has a limited sample size and is restricted to only two regions of Brazil, requiring caution when generalizing the current findings. Second, all data were obtained through the facilitators’ self‐reports, and cross‐sectional data were used, with the facilitators’ measures collected at the same time point. Moreover, data collection methods varied, with two possible alternatives to answering the questionnaires. Some facilitators only agreed to participate by responding to the questionnaire link without direct access to the researcher, which could have influenced the consistency of responses.

Future studies could explore whether facilitators enhance their self‐regulation skills before and after the parenting program training. Also, future studies could examine how individual differences may influence the assimilation of these skills, including whether factors such as prior experience or personality traits can shape facilitators’ self‐regulation. Additionally, research could investigate whether facilitators’ champion behaviors and self‐regulation skills are associated with better parenting outcomes of caregivers who participated in the program. Longitudinal studies examining the potential impacts of these facilitators’ behaviors on the sustainability of the ACT program would also be valuable. Also, a qualitative study could identify the challenges and enablers that the facilitators face in different organizational contexts, supporting implementation and local needs.

In conclusion, the present study reveals that organizational resource availability had an indirect effect on facilitators’ personal champion behaviors through the mediating role of facilitators’ self‐regulation skills in the implementation of the ACT Program. Additionally, the organizational resource availability predicted facilitators’ self‐regulation and experience with program delivery. These findings highlight the significant role facilitators’ behaviors play in disseminating and scaling parenting programs in real‐world settings. Furthermore, this work provides insights into how organizations can support large‐scale program implementation. Additionally, this study contributes to the literature by presenting new evidence on validating one measure for organizational resource availability and two instruments designed to assess key facilitator behaviors—self‐regulation and champion behaviors—in the context of parenting program implementation in a middle‐income country.

## AUTHOR CONTRIBUTIONS

**Camila Regina Lotto**: Conceptualization; investigation; formal analysis; visualization; data curation; writing—original draft; writing—review editing. **Elisa Rachel Pisani Altafim**: Conceptualization; funding acquisition; formal analysis; writing—original draft; writing—review editing; supervision; resources; project administration. **Dana Charles McCoy**: Conceptualization; formal analysis; writing—original draft; writing—review editing; supervision. **Maria Beatriz Martins Linhares**: Conceptualization; funding acquisition; formal analysis; writing—original draft; writing—review editing; supervision; resources.

## CONFLICT OF INTEREST STATEMENT

The authors declare no conflicts of interest.

## ETHICS STATEMENT

The study was approved by the Research Ethics Committee of the Clinical Hospital of the Ribeirao Preto Medical School of the University of São Paulo (Ethics approval number: 79410724.3.0000.5440). All participants signed an Informed and Free Consent Form before the data collection.

## Data Availability

The research data are available upon request to the article's corresponding author.
